# Radiographic Outcomes of Conservative and Operative Treatment in Isolated L1 Fractures

**DOI:** 10.3390/medicina59040695

**Published:** 2023-04-01

**Authors:** Andrea Schuller, Stephan Payr, Lorenz Pichler, Thomas Sator, Anna Ploetzl, Britta Chocholka, Thomas M. Tiefenboeck, Kambiz Sarahrudi

**Affiliations:** 1 Department of Orthopaedics and Trauma Surgery, Division of Trauma Surgery, Medical University of Vienna, 1090 Vienna, Austria; 2 Division of Trauma Surgery, LK Wiener Neustadt, 2700 Wiener Neustadt, Austria

**Keywords:** thoracolumbar fracture, kyphosis angle, vertebral, bi-segmental, elderly population

## Abstract

*Background and Objectives*: The adequate therapy of thoracolumbar fractures in the elderly population is still controversially discussed. The aim of this study was to evaluate and compare the results of conservatively and surgically treated younger (≤60a) and elderly patients (>60a) with fractures of L1. *Materials and Methods*: Patients (231) with isolated L1 fractures were included and treated at the University Clinic of Orthopedics and Trauma Surgery, Division of Trauma Surgery, Medical University of Vienna, during the observation period of 2012–2018. *Results*: Conservative treatment led to a significant increase in the vertebral and bi-segmental kyphosis angle in both age groups (young vertebral: *p* = 0.007; young bi-segmental: *p* = 0.044; old vertebral: *p* = 0.0001; old bis-segmental: *p* = 0.0001). A significant reduction in the vertebral angle in both age groups was achieved after operative treatment (young: *p* = 0.003, old: *p* = 0.007). The bi-segmental angle did not significantly improve after surgery in both age groups (≤60a: *p* = 0.07; >60a: *p* = 1.0). *Conclusions*: The study shows that conservative treatment does not seem to be sufficient for a correction of radiological parameters in young and elderly patients. In contrast, operative treatment led to a significant improvement of the vertebral kyphosis angle, without changing the bi-segmental kyphosis angle. These results suggest a greater benefit from operative treatment in patients ≤ 60a than in older patients.

## 1. Introduction

According to recent epidemiological studies, 2/3 of traumatic vertebral fractures occur in the thoracolumbar junction, and most of all, the first lumbar vertebra (L1), accounting for 49% [1,2]. Thoracolumbar fractures show a high incidence in patients between 20 and 40 years of age and in the elderly population over 60 years [3]. 

There are different reasons for this predisposition: on the level of Th11–12, the rigid thoracic spine connects with the more flexible lumbar lordosis. Additionally, the floating ribs (Th11 and Th12) have no direct sternal connection, and therefore have less stability. Further, the thoracic facet joints are limited in flexion and extension and an anterio-posterior translation of the vertebral bodies is blocked. The lumbar vertebral bodies allow more flexion and extension than lateral flexion and rotation [4].

These different ranges of motions and circumstances of stability between the thoracic and lumbar spine explain the increased risk in fractures of the thoracolumbar transition [5]. The indication for conservative or surgical treatment is based on the clinical presentation, age and general condition of the patient [6,7]. Additionally, fracture-specific features, such as the classification and stability, but also the radiological alignment of the spine, play an important role in the decision for adequate fracture treatment [6,7]. The indications for conservative or surgical treatment in this matter is still discussed controversially [7,8]. 

In literature, conservative treatment strategies are described with satisfactory long-term results and can be applied in many cases [9,10]. The exact method of conservative therapy is often poorly mentioned, especially concerning the type of therapeutic measures and the follow-up concept for clinical and radiological evaluation [8,11].

Regarding surgical treatment, the bi-segmental dorsal stabilization using ligamentotaxis for closed reduction is referred to as an adequate procedure for thoracolumbar fractures [12]. It is mentioned that this procedure can effectively correct the kyphosis by indirect reduction techniques, but it is also associated with a high failure rate [12]. The most common deformity resulting from a traumatic thoracolumbar fracture is the post-traumatic kyphosis. For its evaluation, sagittal radiological parameters can be used. An impairment of these parameters may correlate with an unfortunate clinical outcome and the emergence of chronic back pain [13].

The aim of this study was to investigate the treatment of isolated L1 fractures in patients below and over 60 years of age, with regard to the sagittal radiological parameters and the mobility of patients. For evaluation, the vertebral and bi-segmental kyphosis angle were used. 

## 2. Materials and Methods

This study was approved by the Ethics Committee of the Medical University of Vienna (EC-Code: 1095/2017). 

Initially, 1118 patients with L1 fractures were treated at the University Clinic of Orthopedics and Trauma Surgery, Division of Trauma Surgery, Medical University of Vienna, during the observation period of 2012–2018. After application of the inclusion and exclusion criteria, data of 231 patients (demographic data listed in Table 1) with isolated L1 fractures were obtained for the final retrospective analysis. 

The inclusion criteria included the patient being older than 18 years with an isolated L1 fracture, treated either conservatively or treated surgically, with only a percutaneous bi-segmental dorsal stabilization from Th12 to L2 with polyaxial screws, both of them with at least a follow-up of 12 weeks. 

Exclusion criteria were younger than 18 years of age, missing radiological data, multiple vertebrae fractures, any additive surgical procedure, such a kyphoplasty or laminectomy or a combined surgery with anterior stabilization, pre-existing vertebrae fractures in the medical history, polytrauma and initial therapy at another hospital. 

Data were collected concerning age, sex, injury mechanism, sagittal radiological parameters (vertebral and bi-segmental kyphosis angle), therapy (conservative vs. operative) and mobility. The conservative and operative cohort were further divided by age in young (≤60 years) and elderly patients (>60 years), according to the patients’ age at the time of injury. The data collection of co-morbidities was collected when already diagnosed via medical reports from other hospitals or the general practitioner. Fracture morphology was evaluated according to the AO-spine classification, which represents the established standard [14]. Conservative treatment consisted of the application of a corset for 12 weeks. Operative treatment included percutaneous bi-segmental dorsal stabilization with polyaxial screws of L1. Indications for surgery were: height reduction >25%, instability, canal stenosis or a combination of pathologies. Instability was further defined when radiological signs, such as the widening of the interspinous and interlaminar distance, translation of more than 2 mm, kyphosis of more than 20, dislocations, height loss of more than 50%, and articular process fractures, were present [15]. The radiological outcome was evaluated regarding the choice of treatment and patient’s age. The mean follow-up period was 4.3 ± 6.2 months.

Surgical procedure:

In intubation anesthesia, after preoperative administration of intravenous antibiotics, patients were brought into prone position on a radiolucent carbon table. Then, the closed reduction of the fracture by applying hyperlordosis of the lumbar spine was performed. In case of the anterior edge height being adequately restored and the spinal canal free from compromising fragments, the bi-segmental percutaneous dorsal stabilization was indicated. Then, surgical washing and sterile draping of the surgical area were carried out in the typical manner. The pedicles of Th12 and L2 were identified under image converter control, and the Jamshidi needle was inserted into the pedicles, along with the blunt guide wires into Th12 and L2. The correct position of the guide wires was then verified intraoperatively by a 3D scan. Subsequently, percutaneous placement of four polyaxial pedicle screws was performed. Via the targeting device, two additional incisions of approximately 2 cm proximally were placed for the insertion of the rod. The two rods were pre-bent for additionally applying further lordosis to the fracture. The percutaneous threading of the rod and locking of all four screws was performed. Intraoperative X-ray images were taken, then extensive irrigation of the wound site, skin closure with single button sutures and sterile dressing was followed. The patient was transferred, still intubated, to the postoperative CT scan to verify the obtained results and implant position.

**Figure 1:**
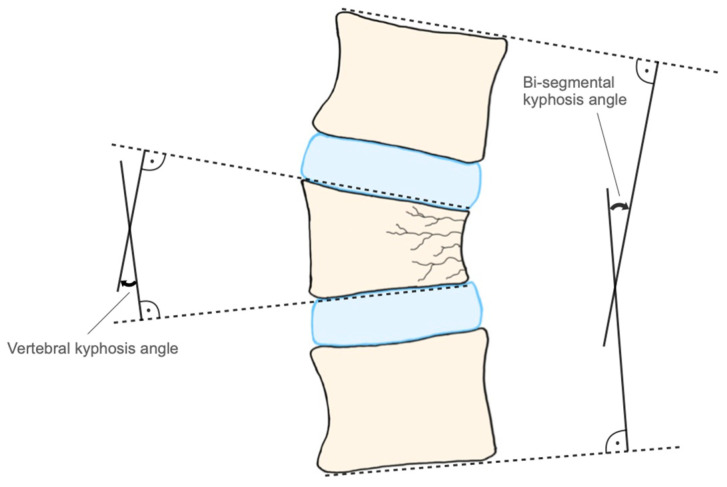
Vertebral and bi-segmental kyphosis angle.

The vertebral kyphosis angle was measured from the cranial and caudal end plate of the fractured vertebra. The bi-segmental kyphosis angle was measured from the caudal endplate of L2 to the cranial end plate of Th12 (Figure 1). This angle includes two segments with the adjacent intervertebral discs. Therefore, a norm value of 7.5° had to be considered for the calculation of differences, since the physiological angle in the lumbar lordosis is 5–10° per se. In this way, a postoperative loss of correction that represents mainly in the intervertebral space can be detected [9]. The radiological measurements of kyphosis angles were performed with the initial and the final follow-up radiographs. Clinical outcomes were evaluated by recording the postoperative mobility. Mobility was assessed at the physical examination and divided into 5 groups: 1: mobile; 2: with aids; 3: rollator; 4: wheelchair; 5: in bed. It was assessed if deterioration in mobility was present by comparing the mobility at the latest follow-up compared with mobility before the injury. 

### Statistical Analysis

Statistical analysis focused on radiological and functional outcomes after treatment of L1 fractures. Clinical and demographic variables (sex, age and follow-up) were examined. 

Statistical analysis and graphic illustration were performed with Graph Pad Prism 6 (GraphPad Software, San Diego, CA, USA). 

Metric-scaled values were tested for normal distribution with the Shapiro–Wilk test, and then compared with a two-sided *t*-test. Not-normally distributed variables were tested with a Mann–Whitney U test. Results were reported as the mean ± standard deviation. Alpha error was set at 0.05. Bonferroni–Holm correction was performed to prevent alpha error accumulation. Statistical significance was set for *p* < 0.05.

## 3. Results

**Figure 2:**
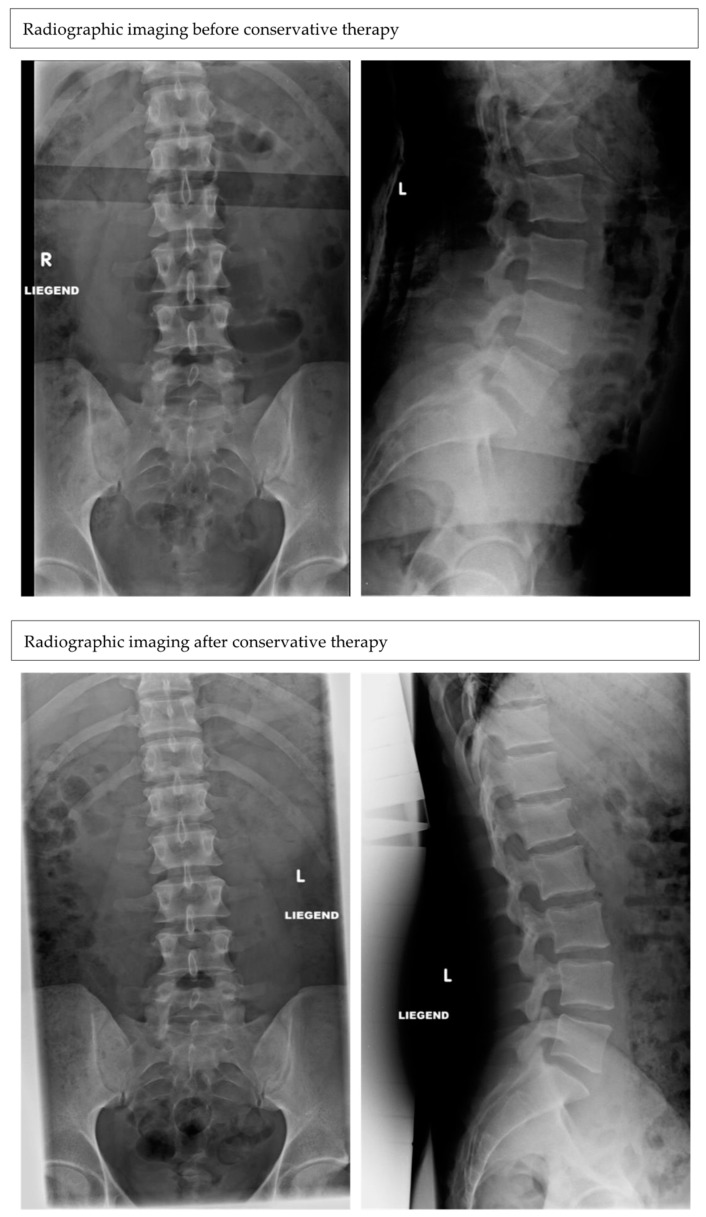
Radiographs (anterior–posterior and lateral) of a patient, 26 years old, with an A1 fracture of L1 after a fall of 2 m, before and after conservative treatment with a brace for 12 weeks in total. No dynamic and further sintering were detected after the last follow-up.

**Figure 3:**
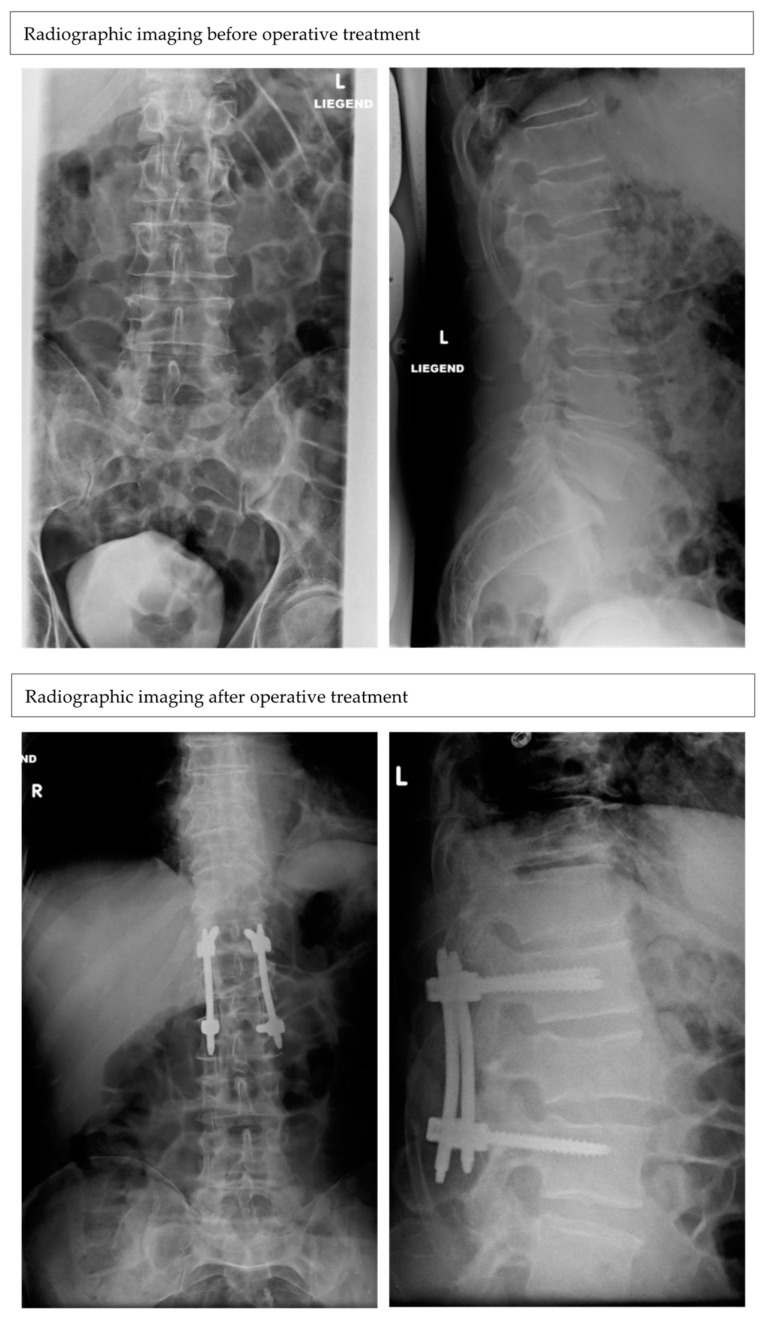
Radiographs (anterior–posterior and lateral) of a patient, 62 years old, with an A3 fracture of L1 after a car crash, before and after operative treatment with a dorsal stabilization. Postoperative reduction was still present after the last follow-up.

In both age groups (young: n = 78 patients, mean age: 41.4 ± 13.5; old: n = 153 patients, mean age: 79 ± 8.8), the most common mechanism of injury for an isolated L1 fracture was the fall from stand (young: 29; 36.7%; old: 136; 88.8%). In the young population, other common injury mechanisms included fall from heights (>3 m) (8; 10.2%), motor vehicle accidents (11; 14.1%) and sports injuries (19; 24.3%). Fracture morphology according to the AO-classification was similar in both age groups. The most common fracture types were the A1 (200 in total) (young: 60; 25.9% vs. old: 140; 60.6%) and A3 (in total 25) (young: 15; 6.4% vs. old: 10; 4.3%). In this study 33 patients were treated operatively (young: 20; mean age: 41.5 ± 12.6 vs. old: 13; mean age 71.3 ± 7.3) and 198 patients conservatively (young: 58; 29.3%, mean age: 41.4 ± 13.5 vs. old: 140; 70.7%, mean age: 79 ± 8.8) (Figure 2 and Figure 3). Indications for surgery were height reduction of the L1 vertebra over 25% (15×), an instable fracture (12×), combination of height reduction and instability (3×) or height reduction and spinal canal stenosis (3×).

The two major risk factors for isolated L1 fractures in the old group were osteoporosis (44 patients; 28.8%) and diabetes mellitus (17 patients; 12.7%). In the young group it was equally osteoporosis and alcohol (6 patients; 7.5%). In the surgical group risk factors were distributed as the following: in the old group osteoporosis, 10 patients; and diabetes mellitus four patients; and two patients with Mb. Ankylosing spondylitis. In the young group, it was one patient each with osteoporosis, obesity and Mb. Ankylosing spondylitis.

The analysis of the sagittal parameters showed an increase in the vertebral and bi-segmental angle, resulting in an impairment in both age groups after conservative treatment (young vertebral: *p* = 0.007; young bi-segmental: *p* = 0.044; old vertebral: *p* = 0.0001; old bis-segmental: *p* = 0.0001).

The results of the operative treatment were as follows: a significant reduction the vertebral angle was achieved in both age groups (young: *p* = 0.003, old: *p* = 0.007). The bi-segmental angle did not significantly improve in both age groups after surgery (young: *p* = 0.07; old: *p* = 1.0). All angles (means ± SD of pre- and post-op) and *p*-values are enlisted in Table 2.

In the operative treatment group, 6/231 patients (2.6%) presented with neurological deficits. In 5/6 patients, these symptoms were recovered from after surgery. In the conservative group, two (0.9%) patients presented with neurological deficits, both fully recovered until the end of treatment.

Complications (such as skin defects and hematoma) occurred in the conservative group, in 8/198 patients (4.0%). In the operatively treated group, 13/33 patients (36.4%) developed a complication including neurological deficits (three patients, 9.1%), screw loosening or breakage, postoperative infection and leakage of cement. In total, four patients (1.7%) had deceased results due to severe comorbidities during conservative treatment (young: 2; old: 2). 

Intermediate deteriorations of mobility were frequently observed, but these improved back to the baseline by the time of the last check-up.

Mobility loss was observed more frequently in the older patient group than in the younger group, measured by percentage. Mobility loss occurs most frequently in older patients after surgical therapy (18%) (Table 3). 

## 4. Discussion

Data of this study show that the conservative treatment of an isolated L1 fracture is not able to correct sagittal parameters in younger and elderly patients. This finding seems to be less surprising and can be explained by the missing ventral support when applying a corset. Much more interesting is the fact that even dorsal bi-segmental fixation, with four polyaxial screws, does not lead to an improvement of the bi-segmental parameter in both age groups. In the literature, the presented results indicate that surgically treated patients have significantly better radiological outcomes, but these findings are presented in patient cohorts with a mean age of approximately 40 years and clinical results are presented to be equal [16]. Still this finding can be brought into accordance with the current literature, which suggests that patients over an age of 50 years of age are predominantly at risk of developing a post-traumatic kyphosis after a vertebra fracture of the thoracolumbar spine [13]. 

Daniaux et al. assumed that degenerative changes in the spine, and therefore limited compensation of the adjacent vertebral segments are a major cause for the ongoing impairment of the posttraumatic kyphosis in the elderly population [17]. 

Additional reasons for the aggravation of the post-traumatic kyphosis could be the short distance of dorsal stabilization and also the use of polyaxial screws, which both the groups had in common [18]. In the younger (≤60a) age group of this study, angles of kyphosis parameters were beneficial in the operative group, accordingly with the literature [19].

The authors suggest that poor bone quality, the higher extent of degenerative changes and the age-related reduction of muscle mass that functions as a kind of dynamic corset–as described in the literature–could additionally explain the issue of a minor improvement of sagittal angles and the observed loss of mobility, especially in older patients after operative treatment [17]. This observed loss of mobility, especially in older patients after operative treatment, may also be caused because of the assumed increased postoperative pain and prolonged physiotherapy, but these considerations are only speculations and could not be illustrated by obtained data.

Despite various treatment options for thoracolumbar fractures, controversial results are presented by different authors [20,21,22,23,24]. Therefore, it still remains unanswered if conservative or operative treatment is more effective [25]. The presented parameters used to determine the evaluation of the final results (radiological, clinical and functional) have ignited an increasing debate about the preferred method for the treatment of thoracolumbar fractures. Although some studies included clinical outcome measures such as pain and functioning, many studies focused on radiological outcomes only, as did this study. It can be criticized that radiological changes are only relevant if being strongly associated with changes in clinical outcomes, but this is not necessarily, true according to van der Roer et al. [25]. Radiological parameters and their measurement of angulation, especially on the sagittal plane of the fractured vertebral segment, has been extensively used for treatment evaluation and postoperative follow-up of patients and most clinical studies have been unable to establish a connection between the degree of kyphosis of the operated vertebral segment, lumbar pain and functional limitation [2,23,24,26]. 

The distribution of the fracture types, according to the AO-classification, is similar to the literature, representing the A1 as the most common [13]. The relatively large number of A1 fractures resembles the mechanism of a low energy trauma. Since in this study only isolated L1 fractures have been included this distribution was expected. 

Despite inferior radiological parameters in the group of elder patients, we did not observe deterioration of their mobility as often as was observed in the conservative group. This may be due to the morphology of the fracture itself, the large number of A1 fractures, and in many cases, due to the initially lower baseline activity level of the elder population. 

Another limitation of this study may be that only two radiological parameters have been investigated, but both of them are highly favorable for the following reasons: the vertebral kyphosis angle is valid to evaluate the fractured vertebra. The bi-segmental kyphosis angle is highly recommendable, because it includes two segments plus their intervertebral discs, and therefore measures the postoperative loss of correction that mostly appears in the intervertebral space. These two parameters show a low intra- and interobserver variability, and therefore a high comparability for studies. Consistent with the literature, data of this study show that the applied parameters are valid for the evaluation of the progress monitoring, as well as the structural success of therapy for fractures of the thoracolumbar region [27]. Additionally, our study does not include patient-reported outcome measures or clinical outcomes, apart from the information of eventual changes of mobility, which is a major limitation. Progressive segmental kyphosis is of particular interest, because it can result in deteriorating pain and progressive deformity. Despite this correlation, the correlation between functional outcomes and radiographic kyphosis has been unclearly defined in the literature. Therefore, we recommend future studies to evaluate clinical outcome measures in comparison to kyphotic changes, to identify what extent of kyphosis results in clinically significant consequences.

The results suggest that both treatment options for patients over 60 years of age seem to be insufficient for the correction of sagittal radiological parameters. Since the costs of therapy are higher in the operative group and the proportion of elderly people in the general population, and thus also in this distinct patient population will continue to grow in the future, further prospective studies, with a longer follow-up and a combination of clinical and radiological outcomes, are recommended in order to be able to suggest therapy guidelines.

However, since the older patient group showed a deterioration of the radiological and mobility outcomes even under conservative therapy, a suggestion to effectively deal with the increasing number of thoracolumbar fractures in the future would be to start prevention already in trauma surgery outpatient departments. By recommending a bone density measurement, including a brochure with subsequent discussion of the findings at the general practitioner’s office, risk factors could be eliminated and awareness could be created. This recommendation should be given to all patients with fractures of the thoracolumbar junction and increased risk, who have been diagnosed in a trauma surgery outpatient clinic. Apart from the fracture classification, osteoporosis has been associated with progressive kyphosis and bone quality may be an important variable in the prediction of progressive kyphosis, since the anterior vertebra column is to be relevant for stability [28]. Further, the literature described in a multivariate logistic regression that osteoporosis is a strong predictor for an unfavorable radiological outcome, including instrumentation failure or abnormal thoracolumbar alignment [29]. It is estimated that in postmenopausal women, 80% remain without adequate therapy after fragility fractures [30]. Even patients who have already been diagnosed with osteoporosis often remain uninformed and untreated [31]. Further risk factor awareness should be created in order to enable the prevention and adequate therapy [32]. Even though the recommendation of osteoporosis screening for patients over 50 years of age with a low-traumatic fracture already exists, no steps in this direction have been taken yet in trauma surgery outpatient departments [33]. In conjunction with the results of this study, the introduction of preventive measures in trauma surgery outpatient departments would be an important step in dealing with the future increase of fragility fractures.

## 5. Conclusions

Summarizing, this study shows the challenge of treating a L1 fracture in the elderly patient group. The reason is that neither conservative nor operative treatment leads to an overall improvement in radiological parameters. The clinical relevance of this study entails a correction of radiological parameters in elderly patients, the conservative as well as the operative treatment with a bi-segmental dorsal stabilization, though they do not seem to be sufficient. 

## Figures and Tables

**Table 1:** Demographic data of patients according to age and treatment.

	Young	Elderly	Operative	Conservative
Number of patients	78	153	33	198
Sex				
Female, n (%)	37 (47.4)	100 (65.4)	21 (63.6)	120 (60.6)
Male, n (%)	41 (52.6)	53 (34.6)	12 (36.4)	78 (39.4)
Age (mean ± SD)	41.4 ± 13.5	79 ± 8.8	52.8 ± 19.7	67.0 ± 19.4
Mechanism of injury				
Fall from stand	29 (37.2)	136 (88.8)	23 (69.7)	142 (71.7)
Fall from height	8 (10.3)	2 (1.3)	8 (24.2)	2 (1.0)
Traffic accidents	21 (26.9)	7 (4.6)	9 (27.3)	19 (9.6)
Sport injuries	19 (24.4)	0	2 (6.0)	17 (8.6)
AO-Classification				
A1	60 (76.9)	140 (91.5)	21 (63.6)	179 (90.4)
A2	0	1 (0.7)	0	1 (0.5)
A3	15 (19.2)	10 (6.5)	18 (54.5)	7 (3.5)
A4	2 (2.6)	1 (0.7)	3 (9.1)	0
B2	1 (1.3)	0	1 (3.0)	0
B3	0	1 (0.7)	1 (3.0)	0
Risk factors				
Osteoporosis	6 (7.7)	44 (28.8)	11 (33.3)	38 (19.2)
Diabetes	6 (7.7)	17 (12.7)	4 (12.1)	19 (9.6)
Mb. Ankylosing spondylitis	2 (2.6)	3 (2.0)	3 (9.1)	2 (1.0)

**Table 2:** Mean ± SD of kyphosis angles in grade, in both age groups, pre- and post-treatment and *p*-values of kyphosis angles pre- vs. post-treatment in both age groups.

	Vert. Pre-Op	Vert. Post-Op	*p*-Value	Bi-Seg. Pre-Op	Bi-seg. Post-Op	*p*-Value
Conservative young (mean ± SD)	6.73 ± 4.07	9.65 ± 4.54	0.007	6.06 ± 5.43	7.81 ± 5.86	0.044
Conservative old (mean ± SD)	7.37 ± 5.21	10.19 ± 5.64	0.0001	7.99 ± 6.36	10.34 ± 6.91	0.0001
Operative young (mean ± SD)	9.59 ± 4.95	3.27 ± 2.84	0.003	10.00 ± 5.87	5.72 ± 4.14	0.07
Operative old (mean ± SD)	7.69 ± 4.0	3.52 ± 3.39	0.007	6.45 ± 5.11	6.24 ± 5.06	1.0

**Table 3:** Number of deteriorations in mobility at latest follow-up compared to mobility before injury.

	Young	Old
Conservative (n = 198)	1 (0.5%)	16 (8%)
Operative (n = 33)	4 (12%)	6 (18%)

## Data Availability

The datasets generated and/or analyzed in the current study are not publicly available due to data privacy, but are available from the corresponding author on reasonable request.
